# In Memoriam

**Published:** 1896-09

**Authors:** 


### IN MEMORIAM—DR. CHAS. G. RAUE.

Dr. Charles G. Raue, the distinguished physician, student, and life-long friend of Hering; the steadfast and faithful follower of the law of Hahnemann, and a great factor in the work of the Homœopathic School for over fifty years, died at his home, 121 North Tenth Street, Philadelphia, Friday, August 21st, 1896. His death is attributed to senile debility, superinduced by overwork, which resulted in paralysis of his mental and physical vigor.

Dr. Raue returned a few weeks previously to his home in this city from a short visit to a son in New Jersey. His illness took on a serious turn soon afterward, and he continued to grow worse very rapidly. He did not take to his bed until several days afterward. He was attended by Dr. J. C. Guernsey, a son of his old friend and associate.

There were gathered around the physician at the time of his death Mrs. Amelia Raue the wife, and his five children, four sons and one daughter—Dr. C. S. Raue, Dr. J. F. Raue, Carl W. Raue, Adolph Raue, and Miss Johanna Raue.

### SKETCH OF A BUSY LIFE.

The death of Dr. Raue, who was a picturesque figure in the world of medicine, an author and writer of wide reputation, removes the last link between the old days of practical Homœopathy, as Hahnemann taught it, and modern Homœopathy as it is expounded at the present day.

He was born in Nieder-Cunnersdorf, a village situated in the vicinity of Loebau, Saxony, on the 11th of May, 1820. He studied in the College of Teachers at Bautzen, from 1837 to 1841, and taught school for several years in Burkau, where he wrote his first work on psychology, *Die Neue Seelen-Lehre Beneckes,* which appeared in 1847, and underwent five editions, the last being printed in 1876. This work was extensively used as a text-book, and has been translated into the English, French, and Flemish languages.

It was at this stage of his life that Dr. Raue determined to enter the field of medicine, a profession for which he had always felt the greatest love and inclination, having even cherished the fond hope in his early youth of some day becoming a physician. The opportunity being offered him to study Homœopathy under Dr. Constantine Hering, he left his native country in 1848 and made Philadelphia his home. Dr. Raue now availed himself of every opportunity to become a through physician; graduated in the Philadelphia College of Medicine in 1850, and gained much knowledge of practical medicine, and especially of the new therapeutics, from his esteemed preceptor and friend, Constantine Hering.

### THE AUTHOR OF MANY BOOKS.

After his graduation Dr. Raue went to Trenton, N. J., where he practiced medicine for several years, but in 1858 he returned to Philadelphia. He at once became actively associated with the new Homœopathic Medical College of Pennsylvania, having been elected to the chair of special pathology and therapeutics as early as 1864. He afterward became a lecturer upon the science of medicine. He was clear, fluent, and instructive. Two years later, owing to a split which took place in the management of the Homœopathic Medical College, the Hahnemann College was founded, Drs. Hering and Raue procuring a charter for this new institution. It became so prosperous that after two years of its existence the old college was merged into it.

Raue's *Special Pathology and Diagnostics With Therapeutic Hints,* made its appearance in 1867, and it is, perhaps, his most widely known work. Besides writing many articles for German and English homœopathic journals and societies, he also edited *The Yearly Record of Homœopathic Literature* from 1870 to 1875, which is a library containing extracts, reviews, and notices of all the important articles which had appeared in the leading homœopathic journals of the world during those years.

In the year 1889, after a life-long study of psychology, in which Dr. Raue has ever sustained that profound interest manifested when he wrote his first work on the subject, *Psychology as a Natural Science,* made its appearance. It was written after much study and personal investigation of the subjects treated of, and has aroused considerable attention and comment in the philosophical world.

Among the medical societies of which Dr. Raue was an active member may be mentioned the American Institute of Homœopathy, of which organization he is a senior member, having been connected with it for over twenty-five years.

He was also an active member of the State and county medical societies, corresponding member to the Homœopathic Institute of Madrid, honorary member of the Alumni Association of the Hahnemann Medical College of Philadelphia, having received the honorary degree from this institution in 1892, and honorary member of the Homœopathic Society of Mexico. He has also been consulting physician to several homœopathic hospitals for many years.—*The Phila. Times.*

---

